# Supplementary material for: Transcriptional heterogeneity of ventricular zone cells in the ganglionic eminences of the mouse forebrain
Source: eLife. 2022 Feb 17;11:e71864. doi: 10.7554/eLife.71864 (PMC8887903; doi:10.7554/eLife.71864)
Supplement: Transparent reporting form [file elife-71864-transrepform1.pdf]

eLIFE
1st Floor
24 Hills Road
Cambridge CB2 1JP, UK
P 01223 855340
W elifesciences.org
T @elife

eLife's transparent reporting form

We encourage authors to provide detailed information *within their submission* to facilitate the interpretation and replication of experiments. Authors can upload supporting documentation to indicate the use of appropriate reporting guidelines for health-related research (see EQUATOR Network), life science research (see the BioSharing Information Resource), or the ARRIVE guidelines for reporting work involving animal research. Where applicable, authors should refer to any relevant reporting standards documents in this form.

If you have any questions, please consult our Journal Policies and/or contact us: editorial@elifesciences.org.

Sample-size estimation

- You should state whether an appropriate sample size was computed when the study was being designed
- You should state the statistical method of sample size computation and any required assumptions
- If no explicit power analysis was used, you should describe how you decided what sample (replicate) size (number) to use

Please outline where this information can be found within the submission (e.g., sections or figure legends), or explain why this information doesn't apply to your submission:

No power analysis was used in this study. For each single cell sequencing experiment, we pooled together brain regions from ≥ 4 embryos. For the E12.5 ganglionic eminences, 3 scRNAseq experiments were performed (1 WT and 2 Nes-dGFP). For the E12.5 Ctx and the E14.5 Ctx/MGE/LGE/CGE samples, 2 scRNAseq experiments were performed (1WT and 1 Nes-dGFP). After quality control analysis was used to remove low quality cells, we analyzed a total of ~60,000 cells from E12.5 and E14.5 embryonic mouse brains. Specific information on sample size, QC parameters and cell numbers can be found in the Methods under the 'Single-cell RNA sequencing and library generation' and 'Single-cell RNA sequencing analysis' sections, as well as Supplementary Figures 1, 2 and 11.

Replicates

- You should report how often each experiment was performed
- You should include a definition of biological versus technical replication
- The data obtained should be provided and sufficient information should be provided to indicate the number of independent biological and/or technical replicates
- If you encountered any outliers, you should describe how these were handled
- Criteria for exclusion/inclusion of data should be clearly stated
- High-throughput sequence data should be uploaded before submission, with a private link for reviewers provided (these are available from both GEO and ArrayExpress)

Please outline where this information can be found within the submission (e.g., sections or figure legends), or explain why this information doesn't apply to your submission:

For each single cell sequencing experiment, we pooled together brain regions from ≥ 4 embryos. For the E12.5 ganglionic eminences, 3 scRNAseq experiments were performed (1 WT and 2 Nes-dGFP). For the E12.5 Ctx and the E14.5 Ctx/MGE/LGE/CGE samples, 2 scRNAseq experiments were performed (1WT and 1 Nes-dGFP). After quality control analysis was used to remove low quality cells, we analyzed a total of ~60,000 cells from E12.5 and E14.5 embryonic mouse brains. Regarding exclusion/inclusion of data, per the standard scRNAseq protocol, cells that did not pass stringent QC measurements (% mitochondria reads, sufficient reads/cell, blood/mural cell contamination from WT samples, >4500 or <1500 genes/cell, etc.) in the scRNAseq datasets were considered outliers and excluded from analysis (as detailed in the Methods and Supplementary Figure 2). When scaling the data, the total number of molecules detected in a cell, % mitochondria reads, and % ribosomal protein encoding gene reads were regressed out. As noted in the 'Data and Software Availability', all of our high-throughput sequencing data is publicly available at the GEO accession number GSE167013. A detailed explanation of everything above can be found in the Methods section of the manuscript.

Statistical reporting

- Statistical analysis methods should be described and justified
- Raw data should be presented in figures whenever informative to do so (typically when N per group is less than 10)
- For each experiment, you should identify the statistical tests used, exact values of N, definitions of center, methods of multiple test correction, and dispersion and precision measures (e.g., mean, median, SD, SEM, confidence intervals; and, for the major substantive results, a measure of effect size (e.g., Pearson's r, Cohen's d)
- Report exact p-values wherever possible alongside the summary statistics and 95% confidence intervals. These should be reported for all key questions and not only when the p-value is less than 0.05.

Please outline where this information can be found within the submission (e.g., sections or figure legends), or explain why this information doesn't apply to your submission:

Descriptions of relevant computational analysis can be found in the figure legends, associated section of the results, or Methods section. More traditional statistical analysis such as precision measures, effect size and p-values are not relevant for the experiments conducted in this manuscript except for the violin plots in Supplementary Figure 7.

(For large datasets, or papers with a very large number of statistical tests, you may upload a single table file with tests, Ns, etc., with reference to sections in the manuscript.)

Group allocation

- Indicate how samples were allocated into experimental groups (in the case of clinical studies, please specify allocation to treatment method); if randomization was used, please also state if restricted randomization was applied
- Indicate if masking was used during group allocation, data collection and/or data analysis

Please outline where this information can be found within the submission (e.g., sections or figure legends), or explain why this information doesn't apply to your submission:

At various points in the manuscript, cells were grouped based on brain region of origin, mouse line of origin, age of mice, or gene expression above a specific threshold. All of these groupings were determined via computational analysis. No masking, blinding or randomization was necessary for this analysis.

Additional data files ("source data")

- We encourage you to upload relevant additional data files, such as numerical data that are represented as a graph in a figure, or as a summary table
- Where provided, these should be in the most useful format, and they can be uploaded as "Source data" files linked to a main figure or table
- Include model definition files including the full list of parameters used
- Include code used for data analysis (e.g., R, MatLab)
- Avoid stating that data files are "available upon request"

Please indicate the figures or tables for which source data files have been provided:

Table 1 provides a list of genes enriched in the VZ, SVZ and MZ cells (related to Figure 3).
